# Supplementary material for: Comparative Analysis of Different Proteins and Metabolites in the Liver and Ovary of Local Breeds of Chicken and Commercial Chickens in the Later Laying Period
Source: Int J Mol Sci. 2023 Sep 21;24(18):14394. doi: 10.3390/ijms241814394 (PMC10531955; doi:10.3390/ijms241814394)
Supplement: Supplementary file 1 [file ijms-24-14394-s001.zip › ijms-2571525-supplementary.pdf]

Table S1. Results of BCA quantification of proteins in liver samples

Serial number	Sample name	Volumes (μL)	Concentration (μg/μL)	Overall amount (μg)
1	G-L-1	350	26.13	9145.5
2	G-L-2	350	21.83	7640.5
3	G-L-3	350	25.66	8981.0
4	H-L-1	350	30.07	10524.5
5	H-L-2	350	25.57	8949.5
6	H-L-3	350	28.82	10087.0

Table S2. Results of BCA quantification of proteins in ovary samples

Serial number	Sample name	Volumes (μL)	Concentration (μg/μL)	Overall amount (μg)
1	G-O-1	550	8.15	4481.4
2	G-O-2	550	20.88	11484.0
3	G-O-3	550	17.93	9861.5
4	H-O-1	550	15.62	8591.0
5	H-O-2	550	10.156	5582.5
6	H-O-3	550	10.2	5610.0

Table S3. Complete list of pathways of upregulated DEPs enriched in the liver between Groups G and H

Group	Protein ID	Gene name	KEGG pathway	Classification
G	F1NB68	TGFB1I1	VEGF signaling pathway	Signal transduction
	A0A1D5PXQ2	SQSTM1	Mitophagy - animal	Transport and catabolism
	A0A089FGZ4	/	Endocytosis	Transport and catabolism
	A0A1D5PXQ2	SQSTM1	Autophagy - animal	Transport and catabolism
	Q98TF5	RPL39	Ribosome	Translation
	A0A3Q2U3X0	LOC101750889	ECM-receptor interaction	Signaling molecules and interaction
	A0A5H1ZRJ7	COL1A2	ECM-receptor interaction	Signaling molecules and interaction
	F1NZZ2	LAMA5	ECM-receptor interaction	Signaling molecules and interaction
	A0A1D5PWN6	COL6A1	ECM-receptor interaction	Signaling molecules and interaction
	A0A1D5PYU1	COL1A1	ECM-receptor interaction	Signaling molecules and interaction
	A0A089FGZ4		Cell adhesion molecules	Signaling molecules and interaction
	F1C6W3	B-LB	Cell adhesion molecules	Signaling molecules and interaction
	O93417	CASP3	TNF signaling pathway	Signal transduction
	A0A1D5PWN6	COL6A1	PI3K-Akt signaling pathway	Signal transduction
	A0A5H1ZRJ7	COL1A2	PI3K-Akt signaling pathway	Signal transduction
	F1NZZ2	LAMA5	PI3K-Akt signaling pathway	Signal transduction
	A0A1D5PYU1	COL1A1	PI3K-Akt signaling pathway	Signal transduction
	O93417	CASP3	MAPK signaling pathway	Signal transduction
	Q90WF1		MAPK signaling pathway	Signal transduction

A0A3Q2UE29	HK2	HIF-1 signaling pathway	Signal transduction
Q6QWE7	ADIPOQ	AMPK signaling pathway	Signal transduction
E1BX85	GSTO2	Glutathione metabolism	Metabolism of other amino acids
Q90940	P22	Riboflavin metabolism	Metabolism of cofactors and vitamins
Q90940	P22	Porphyrin metabolism	Metabolism of cofactors and vitamins
F1N9P5	ABCD4	ABC transporters	Membrane transport
F1NI89	ADH4	Fatty acid degradation	Lipid metabolism
F1NYK6	CBR4	Fatty acid biosynthesis	Lipid metabolism
A0A1D5P6E8	CDH15	Fatty acid biosynthesis	Lipid metabolism
Q5F370	PTGR2	Arachidonic acid metabolism	Lipid metabolism
Q75UT8	pkr	Protein processing in endoplasmic reticulum	Folding, sorting and degradation
E1BZT9	ACSS1B	Methane metabolism	Energy metabolism
F1NI89	ADH4	Methane metabolism	Energy metabolism
E1BZT9	ACSS1B	Carbon fixation pathways in prokaryotes	Energy metabolism
A0A5H1ZRJ7	COL1A2	Focal adhesion	Cellular community - eukaryotes
F1NB68	TGFB1I1	Focal adhesion	Cellular community - eukaryotes
Q90WF1		Focal adhesion	Cellular community - eukaryotes
F1NZZ2	LAMA5	Focal adhesion	Cellular community - eukaryotes
A0A1D5PWN6	COL6A1	Focal adhesion	Cellular community - eukaryotes
A0A1D5PYU1	COL1A1	Focal adhesion	Cellular community - eukaryotes
R4GF71	TMSB4X	Regulation of actin cytoskeleton	Cell motility
F1NB68	TGFB1I1	Regulation of actin cytoskeleton	Cell motility
O93417	CASP3	p53 signaling pathway	Cell growth and death
P08267	FTH	Ferroptosis	Cell growth and death
A0A089FGZ4		Cellular senescence	Cell growth and death
A0A1D5PXQ2	SQSTM1	Cellular senescence	Cell growth and death
O93417	CASP3	Apoptosis - multiple species	Cell growth and death
P07032	ACYP1	Pyruvate metabolism	Carbohydrate metabolism
E1BZT9	ACSS1B	Pyruvate metabolism	Carbohydrate metabolism
F1NI89	ADH4	Pyruvate metabolism	Carbohydrate metabolism
E1BZT9	ACSS1B	Propanoate metabolism	Carbohydrate metabolism
E1BZT9	ACSS1B	Glycolysis / Gluconeogenesis	Carbohydrate metabolism
F1NI89	ADH4	Glycolysis / Gluconeogenesis	Carbohydrate metabolism
A0A3Q2UE29	HK2	Glycolysis / Gluconeogenesis	Carbohydrate metabolism
A0A3Q2UE29	HK2	Fructose and mannose metabolism	Carbohydrate metabolism
A0A3Q2UE29	HK2	Streptomycin biosynthesis	Biosynthesis of other secondary metabolites
A0A3Q2UE29	HK2	Neomycin, kanamycin and gentamicin biosynthesis	Biosynthesis of other secondary metabolites

H	A0A1D5P6E8	CDH15	Valine, leucine and isoleucine degradation	Amino acid metabolism
	F1NI89	ADH4	Tyrosine metabolism	Amino acid metabolism
	E1BVP5	ASPA	Histidine metabolism	Amino acid metabolism
	F1N914	PYCR1	Arginine and proline metabolism	Amino acid metabolism
	E1BSN3	L3HYPDH	Arginine and proline metabolism	Amino acid metabolism
	A0A1L1RR71	ALDH18A1	Arginine and proline metabolism	Amino acid metabolism
	A0A1D5PD08	PNPO	Vitamin B6 metabolism	Metabolism of cofactors and vitamins
	P79760	CYP1A4	Tryptophan metabolism	Amino acid metabolism
	F1NEK9	ACMSD	Tryptophan metabolism	Amino acid metabolism
	A0A1D5P0J6	TDO2	Tryptophan metabolism	Amino acid metabolism
	A0A1L1RP39	UGP2	Pentose and glucuronate interconversions	Carbohydrate metabolism
	A0A1L1RJP8	TUBA4A	Gap junction	Cellular community - eukaryotes
	A0A1D5PD08	PNPO	Biosynthesis of cofactors	Global and overview maps
	F1NEK8	GCH1	Biosynthesis of cofactors	Global and overview maps
	A0A1D5P0J6	TDO2	Biosynthesis of cofactors	Global and overview maps
	A0A1L1RP39	UGP2	Biosynthesis of cofactors	Global and overview maps
	A0A1L1RP39	UGP2	O-Antigen nucleotide sugar biosynthesis	Glycan biosynthesis and metabolism
	Q5ZM72	FAR1	Cutin, suberine and wax biosynthesis	Lipid metabolism
	E7EDS8	FADS1	Biosynthesis of unsaturated fatty acids	Lipid metabolism
	F1NEK8	GCH1	Folate biosynthesis	Metabolism of cofactors and vitamins
	F1P2A6	ADA	Purine metabolism	Nucleotide metabolism
	A0A3Q2U134	GLUL	Two-component system	Signal transduction
	A0A3Q3B2T4	HBS1L	mRNA surveillance pathway	Translation

Table S4. Complete list of pathways of upregulated DEPs enriched in the ovary between Groups G and H

Group	Protein ID	Gene Name	KEGG pathway	Classification
G	A0A3Q2UPM7	PEX11B	Peroxisome	Transport and catabolism
	A0A3Q2TZN9	GABARAPL2	Mitophagy - yeast	Transport and catabolism
	A0A1D5PXV5	SP1	Mitophagy - animal	Transport and catabolism
	A0A3Q2TZN9	GABARAPL2	Mitophagy - animal	Transport and catabolism
	A0A3Q2TZN9	GABARAPL2	Autophagy - yeast	Transport and catabolism
	A0A3Q2TZN9	GABARAPL2	Autophagy - other	Transport and catabolism
	A0A3Q2TZN9	GABARAPL2	Autophagy - animal	Transport and catabolism
	E1BR45	TLN2	Rap1 signaling pathway	Signal transduction
	R4GM98	NME3	MAPK signaling pathway - plant	Signal transduction

	Q4ADJ6	TFEW	HIF-1 signaling pathway	Signal transduction
	A0A3Q2U8J2	G6PC3	AMPK signaling pathway	Signal transduction
	E1BSI0	PARP3	Base excision repair	Replication and repair
	A0A1D5PXN5	TXNRD1	Selenocompound metabolism	Metabolism of other amino acids
	F1NMJ2	AK4	Thiamine metabolism	Metabolism of cofactors and vitamins
	F1NI89	ADH4	Retinol metabolism	Metabolism of cofactors and vitamins
	Q5ZHM4	RCJMB04_35g11	Pantothenate and CoA biosynthesis	Metabolism of cofactors and vitamins
	A0A1D5PHB6	DHX36	RNA degradation	Folding, sorting and degradation
	Q5ZJV9	CNOT7	RNA degradation	Folding, sorting and degradation
	F1N986	CA5A	Nitrogen metabolism	Energy metabolism
	F1NI89	ADH4	Methane metabolism	Energy metabolism
	F1NTM7	GOT1	Carbon fixation in photosynthetic organisms	Energy metabolism
	Q4ADJ6	TFEW	Ferroptosis	Cell growth and death
	A0A3Q2U8J2	G6PC3	Starch and sucrose metabolism	Carbohydrate metabolism
	F1NI89	ADH4	Pyruvate metabolism	Carbohydrate metabolism
	A0A3Q2U8J2	G6PC3	Glycolysis / Gluconeogenesis	Carbohydrate metabolism
	F1NI89	ADH4	Glycolysis / Gluconeogenesis	Carbohydrate metabolism
	A0A3Q2U8J2	G6PC3	Galactose metabolism	Carbohydrate metabolism
	F1NTM7	GOT1	Tropane, piperidine and pyridine alkaloid biosynthesis	Biosynthesis of other secondary metabolites
	F1NTM7	GOT1	Isoquinoline alkaloid biosynthesis	Biosynthesis of other secondary metabolites
	F1NTM7	GOT1	Tyrosine metabolism	Amino acid metabolism
	F1NTM7	GOT1	Phenylalanine, tyrosine and tryptophan biosynthesis	Amino acid metabolism
	F1NTM7	GOT1	Cysteine and methionine metabolism	Amino acid metabolism
	F1NTM7	GOT1	Arginine biosynthesis	Amino acid metabolism
	F1NTM7	GOT1	Arginine and proline metabolism	Amino acid metabolism
	F1NTM7	GOT1	Alanine, aspartate and glutamate metabolism	Amino acid metabolism
	F1NI89	ADH4	Tyrosine metabolism	Amino acid metabolism
	F1NI89	ADH4	Alcoholic liver disease	/
	A0A3Q3AW56	GCDH	Benzoate degradation	Xenobiotics biodegradation and metabolism
	A0A1D5NWE6	LOC107050229	Lysosome	Transport and catabolism
	A0A1D5PU31	PLA2G15	Lysosome	Transport and catabolism
H	F1NJF8	NAGA	Lysosome	Transport and catabolism
	E1C234	SUMF1	Lysosome	Transport and catabolism
	F1NZK8	EFL1	Ribosome biogenesis in eukaryotes	Translation
	F1P2L5	LSG1	Ribosome biogenesis in eukaryotes	Translation
	F1NJT4	FN1	ECM-receptor interaction	Signaling molecules and interaction

	P16047	TGFB3	Cytokine-cytokine receptor interaction	Signaling molecules and interaction
	F1NV61	CASP7	TNF signaling pathway	Signal transduction
	F1P204	ABCC1	Sphingolipid signaling pathway	Signal transduction
	A0A3Q2UE75	NSMAF	Sphingolipid signaling pathway	Signal transduction
	A0A3Q2U5L9	RPS6KA1	mTOR signaling pathway	Signal transduction
	P16047	TGFB3	Hippo signaling pathway	Signal transduction
	A0A1L1RZ64	HMGCS1	Terpenoid backbone biosynthesis	Metabolism of terpenoids and polyketides
	F1NJX8	FNTA	Terpenoid backbone biosynthesis	Metabolism of terpenoids and polyketides
	O73888	HPGDS	Glutathione metabolism	Metabolism of other amino acids
	F1NR99	CPOX	Porphyrin metabolism	Metabolism of cofactors and vitamins
	F1P204	ABCC1	ABC transporters	Membrane transport
	A0A1D5PEM6	STS	Steroid hormone biosynthesis	Lipid metabolism
	A0A1D5NWE6	LOC107050229	Sphingolipid metabolism	Lipid metabolism
	A0A1D5PU31	PLA2G15	Glycerophospholipid metabolism	Lipid metabolism
	A0A1D5NWE6	LOC107050229	Other glycan degradation	Glycan biosynthesis and metabolism
	F1NJF8	NAGA	Glycosphingolipid biosynthesis - globo and isoglobo series	Glycan biosynthesis and metabolism
	A0A3Q2UFW3	STX5	SNARE interactions in vesicular transport	Folding, sorting and degradation
	F1NBN1	SEC61A1	Protein export	Folding, sorting and degradation
	A0A1D5NUY6	NDUFC2	Oxidative phosphorylation	Energy metabolism
	Q5F3V2	RCJMB04_6f14	Carbon fixation pathways in prokaryotes	Energy metabolism
	A1E345	F11R	Tight junction	Cellular community - eukaryotes
	F1NJT4	FN1	Regulation of actin cytoskeleton	Cell motility
	A0A3Q2U5L9	RPS6KA1	Oocyte meiosis	Cell growth and death
	F1NV61	CASP7	Apoptosis - multiple species	Cell growth and death
	F1P5S5	ACSS3	Propanoate metabolism	Carbohydrate metabolism
	A0A1L1RP44	RBKS	Pentose phosphate pathway	Carbohydrate metabolism
	Q5F3V2	RCJMB04_6f14	Citrate cycle (TCA cycle)	Carbohydrate metabolism
	A0A1L1RZ64	HMGCS1	Butanoate metabolism	Carbohydrate metabolism
	A0A1L1RZ64	HMGCS1	Valine, leucine and isoleucine degradation	Amino acid metabolism
	A0A3Q3AW56	GCDH	Tryptophan metabolism	Amino acid metabolism
	A0A3Q3AW56	GCDH	Lysine degradation	Amino acid metabolism
	E1C378	HNMT	Histidine metabolism	Amino acid metabolism

Table S5. Complete list of pathways of upregulated DMs enriched in the liver between Groups G and H

Group	Metabolite	KEGG pathway	Classification

	O-phosphoethanolamine	Sphingolipid metabolism	Lipid metabolism
	Taurocholate	Primary bile acid biosynthesis	Lipid metabolism
	Camp	MAPK signaling pathway	Signal transduction
	Camp	Calcium signaling pathway	Signal transduction
	Camp	Apelin signaling pathway	Signal transduction
	Camp	Oocyte meiosis	Cell growth and death
	Cis-7,10,13,16-docosatetraenoic acid	Ferroptosis	Cell growth and death
	Glutathione	Ferroptosis	Cell growth and death
	Pyruvate	Terpenoid backbone biosynthesis	Metabolism of terpenoids and polyketides
	Pyruvate	Pyruvate metabolism	Carbohydrate metabolism
	Dl-a-hydroxybutyric acid	Propanoate metabolism	Carbohydrate metabolism
	Pyruvate	Pentose and glucuronate interconversions	Carbohydrate metabolism
	Pyruvate	Glycolysis / Gluconeogenesis	Carbohydrate metabolism
	Pyruvate	Citrate cycle (TCA cycle)	Carbohydrate metabolism
	L-Arabinono-1,4-lactone	Ascorbate and aldarate metabolism	Carbohydrate metabolism
	Pyruvate	Ascorbate and aldarate metabolism	Carbohydrate metabolism
	Pyruvate	Taurine and hypotaurine metabolism	Metabolism of other amino acids
	Taurocholate	Taurine and hypotaurine metabolism	Metabolism of other amino acids
	Cys-gly	Glutathione metabolism	Metabolism of other amino acids
	Glutathione	Glutathione metabolism	Metabolism of other amino acids
G	Methanesulfonic acid	Sulfur metabolism	Energy metabolism
	Camp	Progesterone-mediated oocyte maturation	Endocrine system
	Camp	Insulin signaling pathway	Endocrine system
	Camp	GnRH signaling pathway	Endocrine system
	4-pyridoxic acid	Vitamin B6 metabolism	Metabolism of cofactors and vitamins
	Pyruvate	Thiamine metabolism	Metabolism of cofactors and vitamins
	Ketoleucine	Valine, leucine and isoleucine degradation	Amino acid metabolism
	Ketoleucine	Valine, leucine and isoleucine biosynthesis	Amino acid metabolism
	Pyruvate	Valine, leucine and isoleucine biosynthesis	Amino acid metabolism
	4-imidazoleacetic acid	Histidine metabolism	Amino acid metabolism
	Anserine	Histidine metabolism	Amino acid metabolism
	Histamine	Histidine metabolism	Amino acid metabolism
	N-acetylhistamine	Histidine metabolism	Amino acid metabolism
	Pyruvate	Alanine, aspartate and glutamate metabolism	Amino acid metabolism
	4-hydroxyphenylpyruvate	Phenylalanine, tyrosine and tryptophan biosynthesis	Amino acid metabolism
	Phenylpyruvate	Phenylalanine, tyrosine and tryptophan biosynthesis	Amino acid metabolism
	Phenylpyruvate	Phenylalanine metabolism	Amino acid metabolism
	Pyruvate	Phenylalanine metabolism	Amino acid metabolism
	Camp	Hedgehog signaling pathway	Signal transduction

H	Camp	Melanogenesis	Endocrine system
	Uridine	Pyrimidine metabolism	Nucleotide metabolism
	Lumichrome	Riboflavin metabolism	Metabolism of cofactors and vitamins
	Lysine	Biotin metabolism	Metabolism of cofactors and vitamins
	Glyceric acid	Glycerolipid metabolism	Lipid metabolism
	Palmitic acid	Fatty acid elongation	Lipid metabolism
	Cis-9-palmitoleic acid	Fatty acid biosynthesis	Lipid metabolism
	Palmitic acid	Fatty acid biosynthesis	Lipid metabolism
	Linolenic acid	alpha-Linolenic acid metabolism	Lipid metabolism
	Serotonin	Tryptophan metabolism	Amino acid metabolism
	3-(2-hydroxyethyl) indole	Tryptophan metabolism	Amino acid metabolism
	3-hydroxyanthranilic acid	Tryptophan metabolism	Amino acid metabolism
	N-acetyl-5-hydroxytryptamine	Tryptophan metabolism	Amino acid metabolism
	Lysine	Aminoacyl-tRNA biosynthesis	Translation

Table S6. Complete list of pathways of upregulated DMs enriched in the ovary between Groups G and H

Group	Metabolite	KEGG pathway	Classification
G	Dl-a-hydroxybutyric acid	Propanoate metabolism	Carbohydrate metabolism
	Glycolate	Glyoxylate and dicarboxylate metabolism	Carbohydrate metabolism
	Pyridoxal phosphate	Vitamin B6 metabolism	Metabolism of cofactors and vitamins
	Pyridoxal phosphate	Thiamine metabolism	Metabolism of cofactors and vitamins
	L-pipecolic acid	Lysine degradation	Amino acid metabolism
H	1-methyl-l-histidine	Histidine metabolism	Amino acid metabolism
	D-sorbitol	ABC transporters	Membrane transport
	Chenodeoxycholate	Primary bile acid biosynthesis	Lipid metabolism
	2-Dehydro-3-deoxy-D-gluconate	Pentose phosphate pathway	Carbohydrate metabolism
	2-Dehydro-3-deoxy-D-gluconate	Pentose and glucuronate interconversions	Carbohydrate metabolism
	D-sorbitol	Galactose metabolism	Carbohydrate metabolism
	D-sorbitol	Fructose and mannose metabolism	Carbohydrate metabolism
	3,4-dihydroxymandelic acid	Tyrosine metabolism	Amino acid metabolism
	1-aminocyclopropanecarboxylic acid	Cysteine and methionine metabolism	Amino acid metabolism

Table S7. Antibody information for western blotting.

Company	Protein	Protein molecular weight (kDa)	Source	Dilution ratio
ZenBio (Chengdu, China)	ADH4	37	Rabbit	1:1000
ZenBio (Chengdu, China)	FADS1	52	Rabbit	1:1000
ZenBio (Chengdu, China)	STS	65	Rabbit	1:1000
ZenBio (Chengdu, China)	HMGCS1	57	Rabbit	1:1000

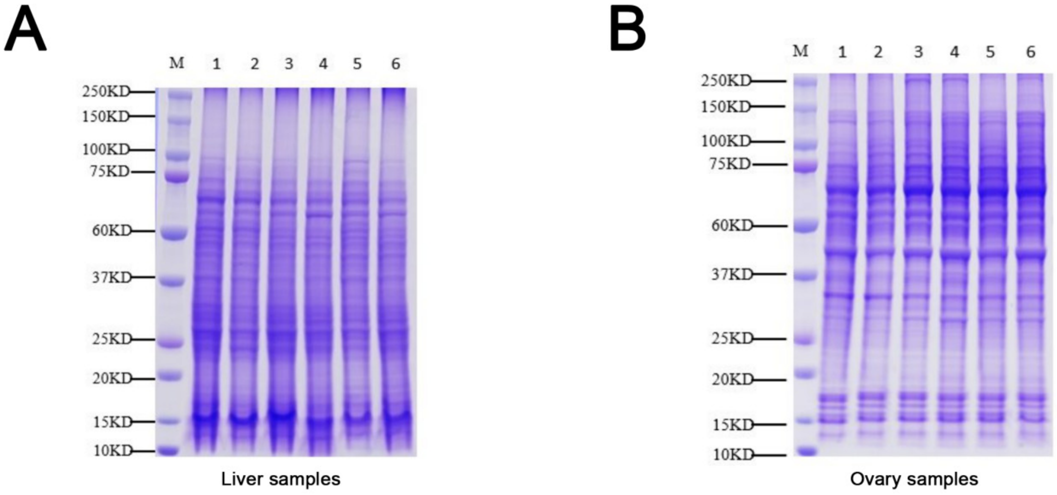

Figure S1. SDS-PAGE results of protein samples from liver and ovary. **(A)** results of SDS-PAGE of proteins in liver samples. **(B)** results of SDS-PAGE of proteins in ovary samples.

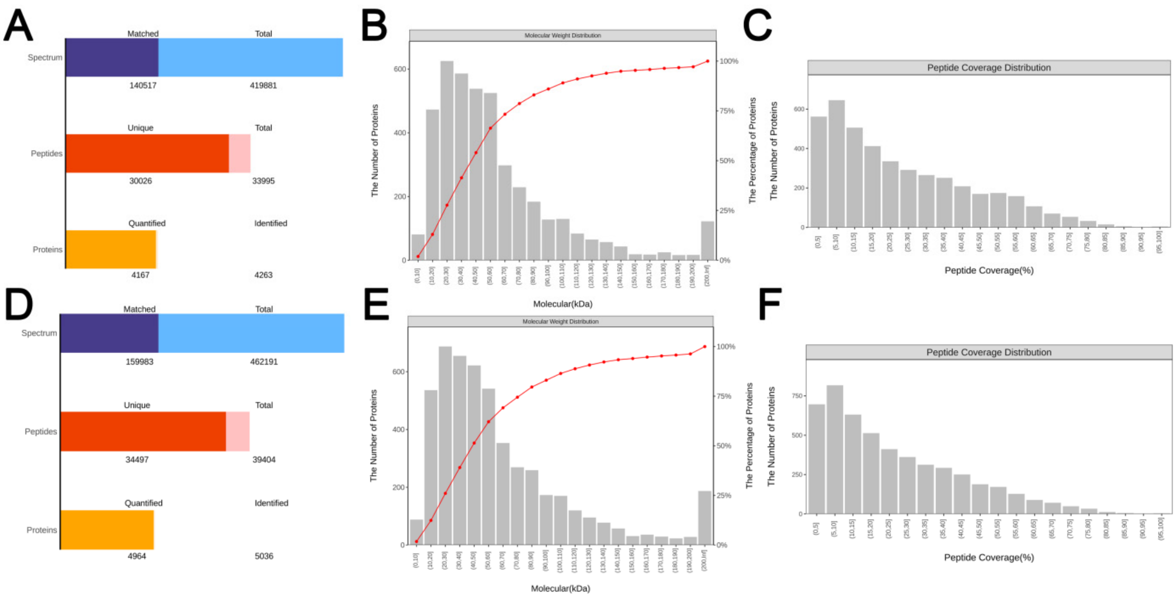

Figure S2. Identification of DEPs in the liver and ovary between Groups G and H. **(A)** identification and quantitative results of proteins and peptides in the liver. **(B)** protein mass distribution in the liver. The X-axis was protein mass interval (kDa), and the Y-axis was the number of proteins. **(C)** protein coverage distribution in the liver. The X-axis showed the percentage coverage interval, and the Y-axis showed the number of proteins. **(D)** identification and quantitative results of proteins and peptides in the ovary. **(E)** protein mass distribution in the ovary. **(F)** protein coverage distribution in the ovary.

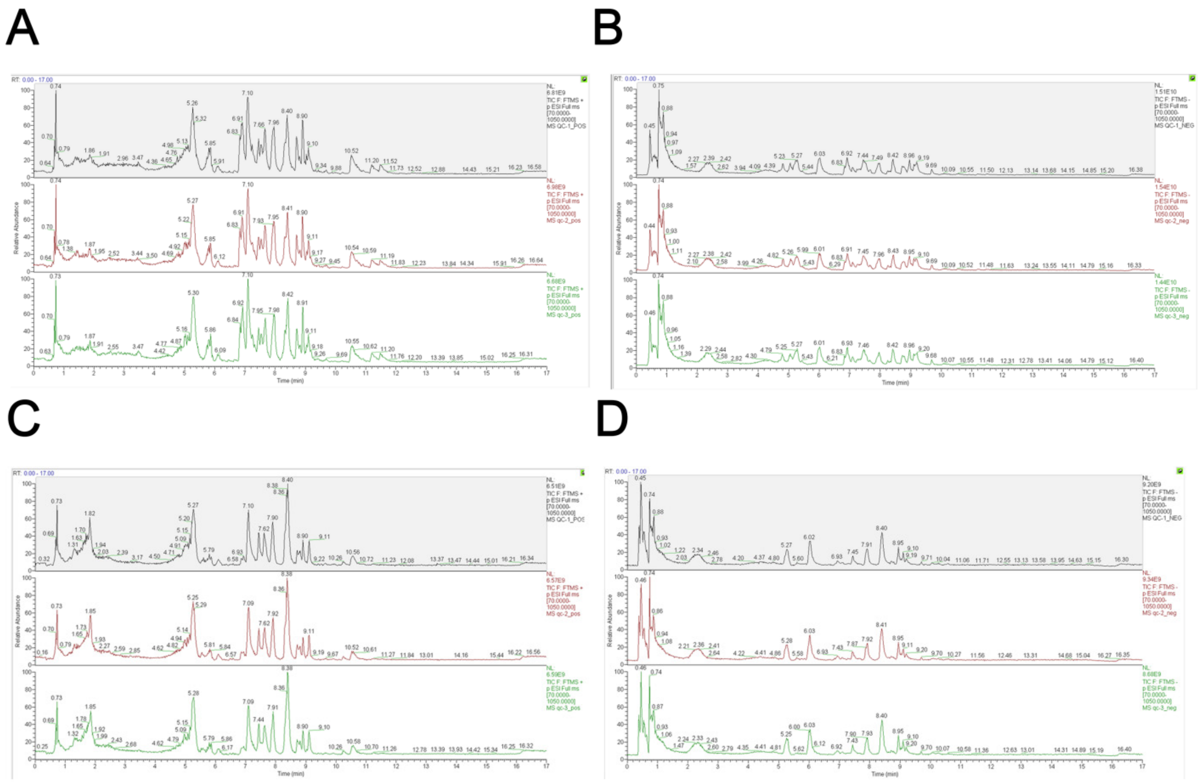

Figure S3. Overlapping spectra of total ion mobility maps of QC samples in positive and negative ion mode. **(A, B)** overlaid spectra of total ion mobility plots of QC samples of liver in positive and negative ion mode. **(C, D)** overlaid spectra of total ion mobility plots of QC samples of ovary in positive and negative ion mode.

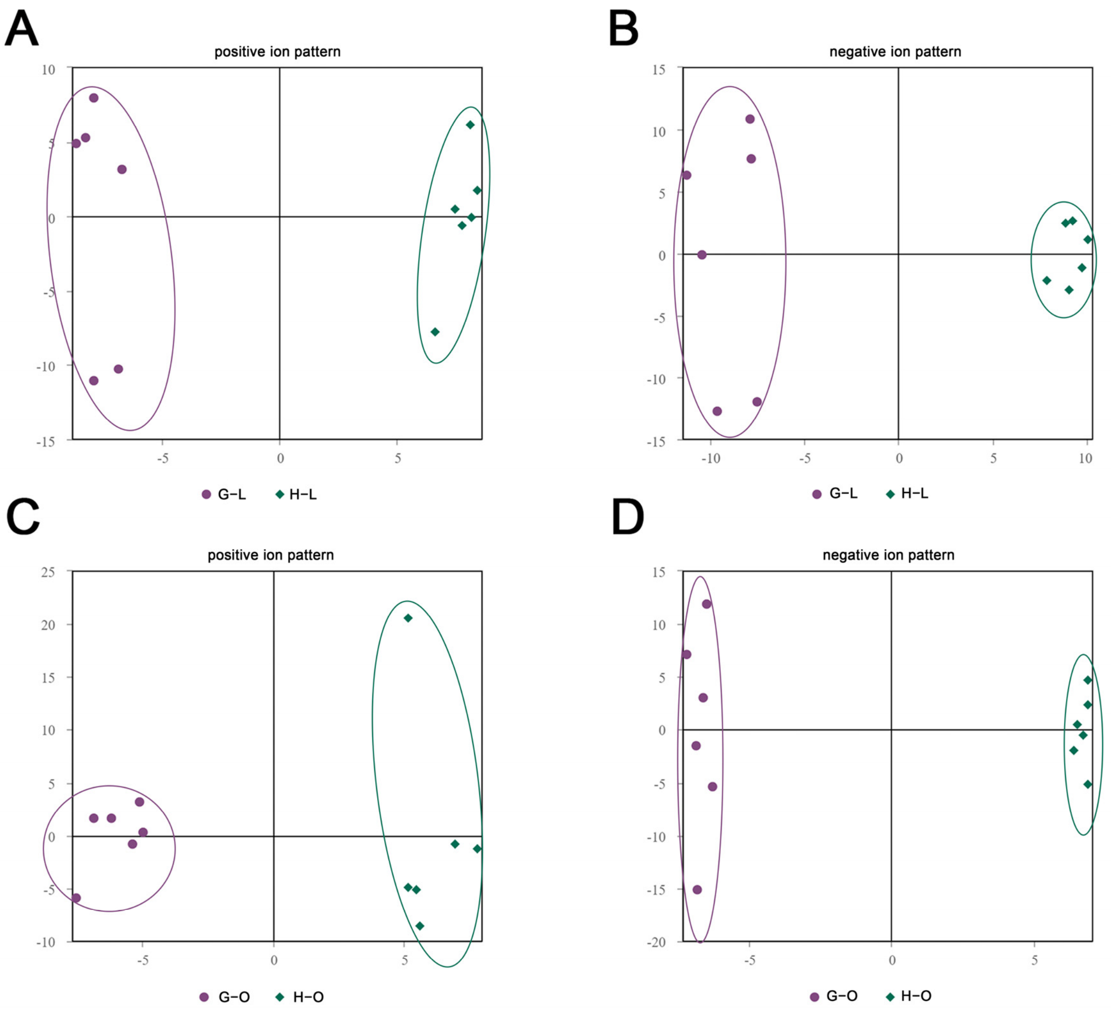

Figure S4. OPLS-DA score plot in positive and negative ion mode. **(A, B)** plot of OPLS-DA scores of liver metabolites in positive and negative ion patterns of Groups G and H. **(C, D)** plot of OPLS-DA scores of ovary metabolites in positive and negative ion patterns of Groups G and H.
